# Macroalgae Has No Effect on the Severity and Dynamics of Caribbean Yellow Band Disease

**DOI:** 10.1371/journal.pone.0004514

**Published:** 2009-02-18

**Authors:** Ivana Vu, Gillian Smelick, Sam Harris, Sarah C. Lee, Ernesto Weil, Robert F. Whitehead, John F. Bruno

**Affiliations:** 1 Department of Marine Sciences, The University of North Carolina at Chapel Hill, Chapel Hill, North Carolina, United States of America; 2 Department of Marine Sciences, University of Puerto Rico, Lajas, Puerto Rico, United States of America; 3 Center for Marine Science, The University of North Carolina Wilmington, Wilmington, North Carolina, United States of America; Northeastern University, United States of America

## Abstract

By removing herbivores and promoting increases in macroalgae, overfishing is thought to indirectly cause coral disease and mortality. We performed three field manipulations to test the general hypothesis that overfishing and the subsequent alteration of coral reef trophic dynamics are a cause of coral epizootics. Specifically, we asked whether the presence of macroalgae can influence within- and among-colony spread rates of Caribbean Yellow Band Disease in *Montastraea faveolata*. Macroalgae were placed next to infected and healthy, adult and small coral colonies to measure effects on disease spread rate, coral growth and coral survival. Surprisingly, the addition of macroalgae did not affect disease severity or coral fitness. Our results indicate that macroalgae have no effect on the severity and dynamics of Caribbean Yellow Band Disease and that fisheries management alone will not mitigate the effects of this important epizootic.

## Introduction

Infectious disease outbreaks are a major cause of coral loss and reef degradation. In the Caribbean, outbreaks of white band disease in the early 1980s nearly extirpated the then dominant species *Acropora cervicornis* and *Acropora palmata* [1]. The white band pandemic led to the regional collapse of coral cover [2], [3] with wide-raging effects on reef inhabitants, geomorphology and ecosystem processes. Evidence from paleontological studies and ecological monitoring indicate that coral disease prevalence, variety, host range, and impacts have increased substantially over the last 30 years [4]–[6].

There are several potential explanations for the observed increase in the severity and impacts of coral diseases. For example, there is evidence that nutrient pollution [7]–[9] and anomalously high ocean temperature [10]–[12] can increase within- and among-colony spread rates of several coral diseases. These and other environmental stressors could increase pathogen virulence and decrease host resistance [13]–[15]. Another widely discussed yet largely untested explanation for increased coral disease is that decades of overfishing [16] have disrupted the balance of coral reef ecosystems, making corals more susceptible to disease outbreaks and other disturbances [17]–[19]. Specifically, the removal of herbivores has led to substantial increases in benthic macroalgae on some reefs [20], which could facilitate disease outbreaks either by acting as pathogen reservoirs or vectors [21] or by increasing the concentration of Dissolved Organic Carbon (DOC)[22].

A recent study found that algae can cause rapid mortality of small coral fragments in closed containers [22]. Related laboratory studies of the effects of DOC on coral health [23], [24] support a potential mechanism through which algae could indirectly cause coral disease outbreaks. Yet many ecologists remain skeptical of a mechanistic link between fishing, macroalgae and coral disease [25], [3], in part due to the paucity of evidence from field experiments.

The purpose of this study was to test the hypothesis that changes in coral reef trophic dynamics and benthic community structure are a cause of increased coral disease severity. Specifically, we asked whether the presence of macroalgae can influence within- and among-colony spread rates of Caribbean Yellow Band Disease (CYBD) in *Montastraea faveolata*, a major reef-building species in the region. We also measured the effects of macroalgae on coral growth and survival. Our results suggest that, at least in these short-term field experiments, macroalgae has no effect on the severity and dynamics of CYBD.

## Methods

### Study Location and System

**Figure 1:**
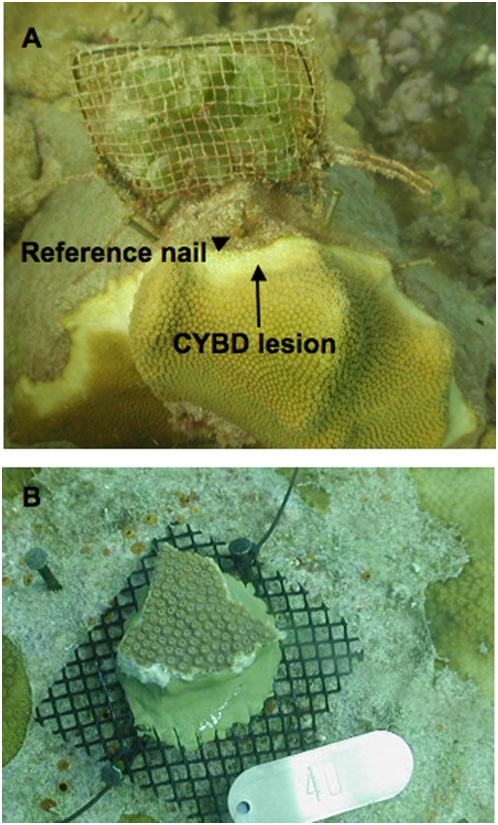
Images of experimental coral colonies. (A) A colony of *Montastraea faveolata* infected with CYBD from experiment 1, treated with a *Dictyosphaeria cavernosa* pouch. (B) An experimental control colony of *Montastraea faveolata* from experiment 3. Photos courtesy J. Bruno.

All experiments were performed *in situ* on Media Luna reef at 8–10 m depth, 1.5 km off La Parguera, on the southwest coast of Puerto Rico during the summer of 2007. Media Luna was chosen because of the high CYBD prevalence (∼25%) and low macroalgal cover (1.4%) (E. Weil, unpublished data). CYBD begins as a small, pale yellowish spot that expands over time to form a yellowish ring around an area of dead coral skeleton [26]–[28] (Fig. 1A). The causative agent of CYBD is reported to be a mix of gram negative *Vibrio* bacteria [29], [30]. Afflicted corals typically have discolored tissue due to the degradation of chlorophyll A pigments [29] and deformed zooxanthellae, suggesting that CYBD is a disease of the symbiotic zooxanthellae [31].

### General experimental design and algal manipulations

All three field experiments included five experimental treatments: a no algae control, a procedural control and three algal addition treatments, each using a different species of macroalgae (n = 12 in experiments 1 and 2, and n = 8 in experiment 3). Experimental algae were collected from nearby reefs and included three locally common species: *Dictyota cervicornis*, *Dictyosphaeria cavernosa* and *Halimeda opuntia*. *Dictyota* and *Halimeda* were included because they are two of the most abundant macroalgae on Caribbean forereefs [32] and were the most common macroalgae at Media Luna when the experiment was performed (E. Weil unpublished data). Additionally, *Halimeda opuntia* is suspected to be a coral disease reservoir or vector [21]. We included *Dictyosphaeria cavernosa*, which is typically a backreef species, because a previous study [22] indicated that it can have strong negative effects on coral health and survival. Algae were added in 12×12 cm mesh pouches (Fig. 1A) filled with a standardized volume (*c.* 300 cm^3^, *c.* 30 g wet algal mass) of algae. Procedural control pouches contained an equivalent mass of plastic mesh but no macroalgae. Pouches were cleaned and replaced with fresh algae every two weeks to prevent algal senescence and a build-up of turf algae and encrusting invertebrates. All experimental host colonies were haphazardly selected, tagged, mapped and randomly assigned one of the five treatments. Colonies for experiments 1 and 2 were intermediate-sized adult colonies ranging in maximum diameter from 26 to 214 cm (92.5 cm±5.1, mean±1 SE, n = 120), spaced >3 m apart. We also measured maximum colony diameter and counted the number of CYBD lesions on each colony for use as covariates in statistical analysis.

### Experiment 1: within-colony lesion advancement

In experiment 1, we measured the effects of algal treatments on within-colony spread rate of CYBD. All colonies in this experiment were already naturally infected with CYBD (as determined by the presence of an active lesion)(Fig. 1A). Macroalgae were placed 3 cm from the trailing edge of yellow band lesions by attaching the mesh pouches to dead portions of the colony with cement nails (Fig. 1A). Treatment effects on the rate of within-colony spread were quantified by measuring yellow band lesion advancement from 3–5 reference nails placed on the dead coral skeleton adjacent to the active lesion (Fig. 1B)(Bruno et al. 2003). The experiment ran for 53 days (May 29 to July 20, 2007) and we measured two aspects of lesion advancement *in situ* to the nearest 1.0 mm every two weeks: (1) distance from the reference nails to the nearest infected but living tissue (i.e., the trailing lesion edge) as a measure of host tissue mortality, and (2) distance from the reference nails to the leading edge of the lesion (i.e., lesion advancement). The average of all replicate measurements for each sampling time on a given colony was used in the statistical analyses.

### Experiment 2: among-colony spread

Experiment 2 was conducted to measure the effects of algal presence on the susceptibility of healthy *M. faveolata* colonies to CYBD. All colonies were initially healthy, i.e., not infected with CYBD or any other known, visible disease. Algal pouches were placed on surrounding substrate ∼3 cm away from the living, healthy tissue of the colony. Infection rate of CYBD was quantified by scoring all colonies as infected or healthy at the end of the 52 day experiment.

### Experiment 3: small colony fitness

The purpose of experiment 3 was to measure *in situ* effects of macroalgae on the growth, survival and CYBD infection rate of small *M. faveolata* colonies. Forty small colonies (n = 8) were collected by removing fragments (mean size = 14 cm^2^, range = 10–25 cm^2^) from large, uninfected colonies with a hammer and chisel. Corals were brought to the nearby Magueyes Island Laboratory, attached to 10×10 cm mesh screens using underwater epoxy, buoyant weighed, photographed and returned to the field within 24 hours. The screens were secured to small patches of cleared substrate using cement nails (Fig. 1B). We then allowed the corals to recover *in situ* for 72 hours before the algal pouches were attached 1–3 cm from the colony edges. After 21 days, all colonies were scored as alive or dead, infected or healthy, and then returned to the lab to be reweighed. Skeletal accretion was measured using the buoyant weighing technique [33]. Calcification was calculated as the increase in skeletal mass normalized by the initial colony surface area. The area of the initial living tissue was calculated from digital images using image analysis software (ImageJ).

### DOC sampling

We quantified the effects of our algal treatments on the concentration of DOC by collecting water samples proximate to experimental corals and algal pouches in experiments 1 and 2 (n = 5/treatment/experiment). Water samples were collected using sterile 30 ml syringes to extract water adjacent to the algal pouch, from the boundary layer over the coral tissue nearest the algal pouch or, for the control colonies, over the approximate center of the colony surface. Corals were approached in a manner so as to avoid disrupting the boundary layer. The syringe was flushed with sample water at the sampling point 2–3 times before collection and care was taken not to sample so close to the colonies that coral mucus was extracted.

The full 30 mL syringes were capped immediately after collection and put on ice until they were processed and refrigerated. All water samples were filtered through acid-washed Supor 0.2 µm filters into 20 mL certified EPA vials with teflon lids, treated with 5 µL of 25% phosphoric acid per mL of sample and refrigerated within three hours of their collection to avoid any DOC-altering biotic activity.

DOC concentration was determined by high temperature combustion (HTC) using a Shimadzu TOC 5050A total organic carbon analyzer equipped with an ASI 5000 autosampler (Shimadzu, Kyoto, Japan) following the precautions of Benner and Strom [34]. Standards were prepared from reagent grade potassium hydrogen phthalate (KHP) in Milli-Q Plus Ultra Pure Water. Samples and standards were acidified to pH 2 with 2 M HCl and sparged with CO_2_ free carrier gas for 5 min at a flow rate of 125 ml min^−1^ to remove inorganic carbon. The samples were injected (75 µL) into the Shimadzu TOC-5050A furnace, filled with a preconditioned Shimadzu catalyst (Al_2_O_3_ impregnated with 0.5% platinum), at 680°C. The combustion products were carried by high purity CO_2_ free air through a Peltier cooler at ∼1°C (electronic dehumidifier) for removal of water vapor followed by a sub-micron particle filter and finally into the Shimadzu NDIR detector cell to measure the CO_2_ generated from the combusted carbon. Each sample was injected 4 times and mean values were used in analyses. A seawater reference sample from the Hansel Laboratory Deep Seawater Reference (Lot # 06-00, Bermuda Biological Station for Research Inc.) was included in each run. The average and standard deviation (n = 4) for the reference sample was 44±1.6 µM C as compared to the accepted value of 44±1.5 µM C [35]

## Results

**Figure 2:**
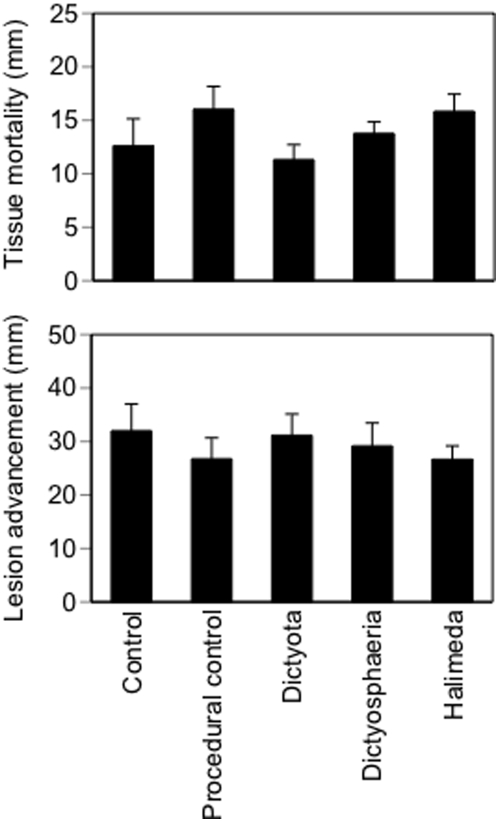
Results of experiment 1. Algal treatment effects on final host tissue mortality and lesion advancement. Values are means±1 SE (n = 12).

In experiment 1 there were no main treatment effects on host tissue loss (Fig. 2; *Treatment* P = 0.307, *Sampling (time)* P = 0.0001, df = 4,55, based on a Repeated Measures ANOVA performed in the Fit Model platform of JMP 6) or lesion advancement (*Treatment* P = 0.115, *Sampling (time)* P = 0.001, df = 4,55). Initial models that included colony size and the number of lesions did not produce qualitatively different results, so these covariates were not included in the final analyses. Power analysis based on a simple one factor ANOVA of only the final sampling found that there was sufficient power (0.99) when δ was set at 1/3 of the mean response. The average rate of lesion advancement across all 5 treatments was 1.65 cm month^−1^, which is ∼3× greater than previously reported values [8].

**Figure 3:**
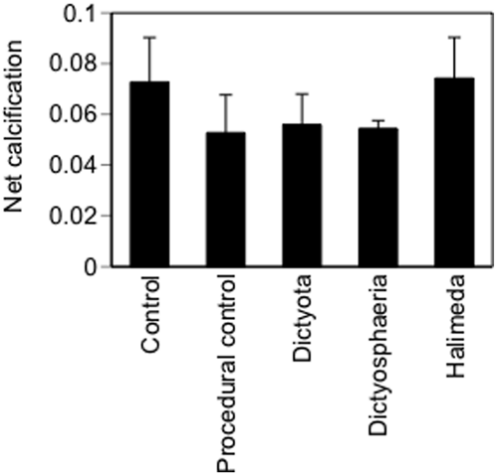
Results of experiment 3. Algal treatment effects on coral calcification (g/cm^2^). The experiment ran for 21 days. n = 8.

In experiment 2, colony survival was 100% in all five treatments. Only 7 of the originally healthy colonies were infected with CYBD during the experiment (Table 1) and the macroalgal treatments had no effect on infection state (Pearson chi-square P = 0.379). In experiment 3, none of the small coral colonies became infected or died, including the 24 colonies treated with macroalgae (Table 1). Thirty-eight of the 40 colonies grew during the experiment and there were no treatment effects on calcification rate (Fig. 3; one factor ANOVA P = 0.69, df = 4,33, Power = 0.69 when δ = 0.02, 1/3 of the mean response).

**Table 1:** Results of experiments 2 and 3.

	Control	P. Control	*Dictyota*	*Dictyosphaeria*	*Halimeda*
Experiment 2					
Mortality	0	0	0	0	0
Infection	25	8.3	8.3	0	16.7
Experiment 3					
Mortality	0	0	0	0	0
Infection	0	0	0	0	0

Effects of algal treatments on percent mortality and infection (%) by CYBD of *M. faveolata* in two field experiments (n = 12 for experiment 2 and 8 for experiment 3).

**Figure 4:**
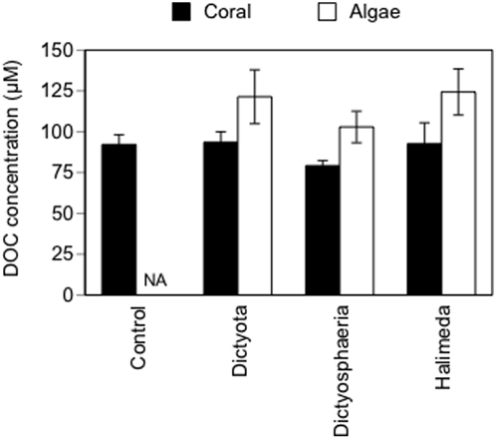
Field measurements of DOC concentration. Concentration of DOC next to the algal pouches and on the surface of the experimental corals. Measurements are pooled from experiments 1 and 2. NA = not applicable (there was no algal pouch for the control treatment). Values are means±1 SE (n = 10).

DOC concentrations did not vary significantly between experiments 1 and 2, so the data were pooled for the final analysis (Fig. 4, n = 10). DOC concentration measured adjacent to the algal pouches did not vary among the three algal species (P = 0.69). DOC concentration adjacent to the coral colonies was significantly lower (mean = 91±5.1 µM C) than concentrations adjacent to algal pouches (mean = 122±8.7 µM C, P = 0.002; comparison excluding control treatments for which algal DOC concentration could not be sampled).

## Discussion

The depletion of large vertebrate consumers relative to their prey has caused a skewing of trophic structure towards dominance at lower levels and the general alteration of food webs [36], [37]. Ecologists strongly suspect that such changes to trophic dynamics, particularly the loss of top predators, will have penetrating effects throughout communities, altering critical ecosystem processes and services [17], [38]. For example, the alteration of coral reef food webs by fishing could decrease community resistance to disease outbreaks and other disturbances [18]. One potential pathway through which fishing could influence coral disease dynamics is by removing key herbivores, thereby increasing the biomass of macroalgae [22].

However, our results do not support the hypothesis that coral disease is caused or exacerbated by macroalgae. We found that the addition of three species of common macroalgae had no detectable effect on any of our measures of CYBD severity or coral fitness. In experiment 1, within-colony lesion advancement and coral mortality were not affected by any of the three macroalgal addition treatments relative to the controls (Fig 2). In experiment 2, macroalgal additions had no effect on CYBD infection rates (Table 1). In fact, control colonies had the highest infection rate (25%), which was 3× greater than the rate observed in the combined algal addition treatments, although this trend was not statistically significant.

Nugues et al. (2004) reported that *Halimeda opuntia* can act as a reservoir for the pathogen responsible for white plague disease. Adding 1000 cm^3^ of macroalgae for 30 days increased the prevalence of white plague in *Montastraea faveolata* to 55% compared to 0 in the control treatment [21]. Yet in our study, across the three experiments, none of the 32 coral colonies treated with *Halimeda opuntia* displayed any signs of white plague infection. The striking difference in outcomes between the two experiments could be explained by the fact that unlike Nugues et al., we did not add the macroalgae directly on top of the healthy coral tissue (i.e., direct contact may be required for successful pathogen transmission).

In experiment 3, none of the 24 small corals treated with macroalgae in the field for 21 days became infected or died. In contrast, Smith et al. (2006) reported that small corals held in plastic containers with macroalgae experienced 100% mortality within 48 hours. Although highly atypical of natural coral-algal interactions, this result was interpreted as evidence that coral diseases can be caused by macroalgae (Smith et al. 2006). No other study pairing small or juvenile corals with macroalgae has reported such striking effects on coral survival. Most similar studies, nearly all of which were performed in the field, have found only small or no effects on coral mortality, particularly when physical contact between corals and algae was precluded [39]–[41]. For example, a recent long-term field experiment found that in the absence of shading and abrasion, the presence of macroalgae had no effect on juvenile coral mortality [41]. This study also found that plastic algal mimics had the same deleterious effects as living macroalgae, indicating that the negative algal effects documented in natural settings are due to abrasion, shading, overgrowth and other related mechanisms, rather than allelochemicals or other algal exudates such as DOC [39].

The DOC concentrations we measured adjacent to corals and macroalgae were within the range of values reported from other reef locations [24]. Our DOC measurements suggest that all three species of macroalgae did moderately increase DOC concentration (Fig. 4). However, this effect was highly localized and was not detected on the surface of the experimental corals, just 3–5 cm away from the algal pouches, probably due to diffusion. Surprisingly, given the perceived role of DOC in coral disease dynamics, no other study has documented *in situ* effects of macroalgae on DOC concentration on a coral reef. DOC release and the subsequent effect on local and reef-wide benthic DOC concentrations are likely influenced by macroalgae biomass, composition, state (e.g., grazing and other stresses could increase DOC release) and by flow characteristics such as velocity and turbulence. Understanding the role of these and other environmental factors in regulating a mechanistic link between algae and DOC is clearly an important (and neglected) step in understanding what effects macroalgae might have on corals and coral disease via DOC release.

### Conclusion

The genus *Montastraea* is one of the most important groups of corals in modern western Atlantic coral reefs [42] that has dominated portions of Caribbean reefs for at least the past 22 million years [43]. Over the last decade CYBD has become the major factor in the loss of live tissue and colonies in this genus [44] with several measures of severity increasing noticeably over the last five years. Recent observations suggest that; (1) lesion growth rate is increasing, (2) colonies with multiple infections are becoming more common, and (3) outbreaks and prevalence of CYBD have been increasing in many localities throughout the Caribbean [27], [45], [8], [28], [46], [44]. Although environmental factors including ocean temperature [47] and nutrient pollution [8] have been implicated in the observed increased severity of CYBD, our experimental results do not support the hypothesis that macroalgae have played a role.

Given the enormous ecological and societal importance of coral reefs, reversing coral loss is a top management priority [48]. A direct causal link between fishing, macroalgae and coral disease would indicate that coral epizootics could be controlled in part by implementing marine reserves or other fisheries management strategies designed to prevent algal blooms. Clearly under different conditions, at other locations or using more or other species of macroalgae, our results might have been different. But within the context and duration of our three experiments, our results suggest that macroalgae has no effect on the severity and dynamics of CYBD. Therefore, limiting macroalgae is unlikely to reduce the prevalence of CYBD and possibly of other important coral diseases.
